# Supplementary material for: A reinforced lunar dynamo recorded by Chang’e-6 farside basalt
Source: Nature. 2024 Dec 19;643(8071):361–5. doi: 10.1038/s41586-024-08526-2 (PMC12240856; doi:10.1038/s41586-024-08526-2)
Supplement: Supplementary file 1 — The file includes Supplementary Discussions 1–6, Supplementary Figs. 1–4, Supplementary Tables 1–5 and Supplementary References 46–75. [file 41586_2024_8526_MOESM1_ESM.pdf]

nature portfolio

https://doi.org/10.1038/s41586-024-08526-2

Supplementary information

A reinforced lunar dynamo recorded by Chang'e-6 farside basalt

In the format provided by the authors and unedited

Supplementary Material for

A reinforced lunar dynamo recorded by Chang'e-6 farside basalt

Shuhui Cai[1,2*], Kaixian Qi[1,2], Saihong Yang[3], Jie Fang[1,2], Pingyuan Shi[1,2], Zhongshan Shen[1], Min Zhang[4], Huafeng Qin[1,2], Chi Zhang[4], Xiaoguang Li[1], Fangfang Chen[1,2], Yi Chen[1,2], Jinhua Li[2,4], Huaiyu He[1,2], Chenglong Deng[1,2], Chunlai Li[3], Yongxin Pan[2,4] & Rixiang Zhu[1]

[1]State Key Laboratory of Lithospheric and Environmental Coevolution, Institute of Geology and Geophysics, Chinese Academy of Sciences, Beijing, China.

[2]College of Earth and Planetary Sciences, University of Chinese Academy of Sciences, Beijing, China.

[3]Key Laboratory of Lunar and Deep Space Exploration, National Astronomical Observatories, Chinese Academy of Sciences, Beijing, China.

[4]Key Laboratory of Earth and Planetary Physics, Institute of Geology and Geophysics, Chinese Academy of Sciences, Beijing, China.

[*]e-mail: *caishuhui@mail.iggcas.ac.cn*

Supplementary Discussion

1. Palaeointensity

1.1 Palaeointensity experiment

The non-thermal palaeointensity method is widely used for measuring extraterrestrial samples since it is nondestructive and can avoid thermal alteration during heating. In this study, we employed the anhysteretic remanent magnetization (ARM)- and isothermal remanent magnetization (IRM)-correction methods[23,24]. Ratios of the alternating field (AF) demagnetization spectrum of natural remanent magnetization (NRM) to those of ARM and IRM are used to calculate palaeointensities of the samples in these methods. The non-thermal palaeointensity experiment steps are as follows:

(i) NRM of the sample is AF demagnetized and measured until 150 mT with intervals of 1–5 mT.

(ii) An ARM is imparted with a direct current (DC) field of 0.05 mT and a peak AF field of 150 mT, and then AF demagnetized and measured with the same intervals as in step (i).

(iii) IRM of the sample is imparted in a field of 1 T and AF demagnetized following the procedure in step (i).

For extraterrestrial materials, the samples may acquire a gyroremanent magnetization (GRM) during AF demagnetization, which will cause bias in the demagnetization results, especially for samples with weak magnetic signals[46]. Therefore, GRM correction was incorporated in the NRM AF demagnetization procedure following the Zijderveld-Dunlop method[47], where the remanence after AF demagnetization along axes x, y, and z was measured respectively and then averaged to eliminate the GRM acquired at different orientations. This cycle was repeated once at high alternating fields to reduce arbitrary noise.

The calculation formulas are $B_{anc} = (1/f') \times (\Delta NRM/\Delta ARM) \times B_{lab}$ for the ARM-correction method[23] and $B_{anc} = a \times (\Delta NRM/\Delta IRM)$ for the IRM-correction method[24], where B_{anc} is the ancient field intensity producing the NRM, B_{lab} is the DC bias field for the ARM, ΔNRM is the NRM lost in step (i), ΔARM is the ARM lost in step (ii), and ΔIRM is the IRM lost in step (iii); f' and a are calibration factors. The single values of NRM versus ARM and IRM after cleaning the secondary overprint were also used for palaeointensity calculation, which are referred to as the AREMc (ref. [14]) and REMc (ref. [48]) methods, respectively. An advantage of the methods is its ability to estimate the palaeointensity for samples that have secondary overprints and suffer from large noise caused by spurious ARM and/or GRM during the AF demagnetization in the high-field range. The calculation formulas are $B_{anc} = (1/f') \times (NRM_{LC}/ARM_{LC}) \times B_{lab}$ and $B_{anc} = a \times (NRM_{LC}/IRM_{LC})$, where B_{anc} is the ancient field intensity, NRM_{LC}, ARM_{LC}, and IRM_{LC} are the residual NRM, ARM, and IRM after

cleaning the low-coercivity overprint. *f'* and *a* are the calibration factors. We adopted the widely used empirical values of *f'* = 1.34 and *a* = 3,000 μT in the modern lunar palaeointensity studies[23,24].

1.2 Palaeointensity results

The non-thermal ARM and IRM palaeointensity results are shown in Figure 3 and Extended Data Figures 1–3, and summarized in Extended Data Table 1. All the samples contain three components roughly after the NRM decay. Sample 211 exhibits an origin-trending high coercivity (HC) component after 50 mT with a maximum angular deviation (MAD) of 30.9° and a deviation angle (DANG) of 8.6° (Fig. 3). The palaeointensities calculated using the ARM and IRM methods are quite consistent within 1 standard error (s.e.), which are 11.63 ± 2.91 μT and 8.28 ± 2.03 μT, respectively. The uncertainty represents 1 s.e. resulting from the linear regression process. The HC component of sample 038 also shows an origin-trending behaviour after 58 mT (MAD = 25.7°, DANG = 11.2°) with the ARM and IRM palaeointensity estimated to be 8.07 ± 1.00 μT and 5.67 ± 0.52 μT, respectively, which agree with each other within 2 s.e.. Sample 344 has a HC component starting at 26 mT with MAD = 25.9° and DANG = 15.3°, and the corresponding ARM- and IRM-based palaeointensities are 13.49 ± 3.19 μT and 21.29 ± 3.12 μT, respectively. The HC component of sample 392 has a higher DANG (60.3°) compared to its MAD (22.9°), which is considered not to be origin-trending. Although the palaeointensities estimated with the ARM (10.08 ± 1.30 μT) and IRM (9.01 ± 1.11 μT) methods are consistent with those of samples 211 and 038, indicating they are possibly reliable, the results yielded with the AREMc (<20.78 μT) and REMc (<15.96 μT) methods were chosen for further discussion.

1.3 Origin of the magnetization component

The primary cooling rate for the basalt clasts, when they formed as part of a mare basalt flow, is essential for estimating whether the samples could have recorded a transient field such as one resulting from impact plasmas. To assess this possibility, we calculated the primary cooling rate of the basalt clasts in this study according to the grain size and crystal structures of the ilmenite in the samples using the crystal size distribution (CSD) method[49]. The cooling rates of the basalt casts 038, 392, 211, and 344 were estimated to be 106 °C hr^{-1}, 55 °C hr^{-1}, 24 °C hr^{-1}, and 19 °C hr^{-1}, respectively, which probably cooled faster than some of the Apollo mare basalts with reported cooling rates of <3 °C hr^{-1} (refs. [2,50]). However, the results indicate it takes >6 hr, with an average of ~20 hr, for these basalt clasts to cool from the Curie temperature of iron (770°C) to the lunar surface temperature, indicating there is a rather

low possibility that the samples, specifically the HC components, have recorded an impact transient field during the original cooling of the volcanic flow(s).

The directional uniformity of the medium coercivity (MC) components can be tested by comparing the angular differences between the MC and HC components. We calculated the angular differences for each sample, and the results display considerable variations (Supplementary Table 4), indicating the directions of the MC components are nonuniform and thus demonstrating they were acquired after the fragmentation of the basalts. The viscous remanent magnetization (VRM) and IRM tests exclude them from both VRM and IRM contamination (Supplementary Discussion 2), which imply that the MC components were most probably acquired on the Moon. Considering their relatively hard and stable AF demagnetization behaviour and the evidence that they may have experienced limited shock pressure of <5 GPa (Supplementary Discussion 4), it is inferred that they are also unlikely to be a shock remanent magnetization (SRM)[29].

To further constrain the origin, we conducted a thermal demagnetization experiment on a sister specimen cut from sample 211 and named 211b here (Supplementary Fig. 2a). A low temperature (LT) component was demagnetized below 100°C first, and a medium temperature (MT) component from 100–250°C was then isolated, which accounts for a large portion (>80%) of the NRM. After a transitional direction between 250 and 400°C, which is possibly attributed to the interaction between the iron and troilite intergrowth as reported in some of the Apollo and Chang'e-5 basalt samples[17,51], a high temperature (HT) component was revealed after 400°C and lasted until 600°C. The behaviours of the LT, MT, and HT components of specimen 211b correspond well to the LC, MC, HC components of sample 211, indicating that a low temperature heating event producing the MC/MT components may exist. A possible scenario for their origin is that the basalts were baked to a temperature of ~250°C by the heat flux generated by a nearby impact event, and then cooled down in impact-related fields such as an amplified dynamo field or transient field generated by charge separation in the impact plasma cloud[15,32,52].

To verify this scenario, we conducted a numerical simulation to estimate the cooling rate of the basalt clast using COMSOL software. Assuming heat can be transferred by conduction and radiation, we considered four cases assuming that a spherical clast with a radius of 3 mm (mimicking the size of sample 211) was buried in the ground with different depths of 1 mm and 4 mm, and cooled down from 300°C to 100°C with different ambient temperatures of -100°C and 0°C. The heat conductivity, density, specific heat, and emissivity of the basalt were set to 1.75 $Wm^{-1}K^{-1}$, 2,920 kg/m^3, 850 $Jkg^{-1}K^{-1}$, and 0.85, respectively, following refs. [28,53]. The calculated cooling time from 300°C to 100°C ranges from

~5–27 s (Supplementary Fig. 2b), implying there is a chance that the sample could record a partial thermal remanent magnetization (TRM) from a transient field that lasted longer than this cooling time, such as a field related to an impact crater larger than a few kilometres according to the calculation of ref. [32]. This inference is further supported by the existence of km-sized craters that formed after the eruption of the mare basalt at the Chang'e-6 landing site[54,55].

An alternative interpretation of the inconsistent angular differences between MC and HC among the samples could be that both the MC and HC components were acquired after fragmentation of the basalts, and thus neither would be a primary record of the lunar field from cooling. This could be tested by measuring mutually oriented specimens of a sample, which is strongly recommended if applicable. Regrettably, the basalt clasts used in this study are small, at the millimetre scale (Fig. 2), and their NRMs are relatively weak, typically at the order of 10^{-10} Am^2 (Extended Data Figs. 1–3). Therefore, in order to ensure adequate signal-to-noise ratio, each basalt clast was treated as a single sample without mutually oriented subsamples. However, unlike the MC components, the HC components recorded consistent palaeointensities generally, which almost excludes their possibility of recording an impact-related transient field after fragmentation.

1.4 Palaeointensity fidelity limit test

Some lunar samples are probably non-ideal palaeointensity recorders since their dominant remanence carrier may be multi-domain (MD) particles, which are prone to acquiring GRM and spurious ARM, potentially introducing bias in palaeointensity estimations. Estimating the ability of a sample to record a certain magnetic field is essential for explaining the palaeointensity data. Ref. [50] proposed a fidelity test, during which samples are imparted ARMs in a known range of DC fields to mimic the thermal-induced NRMs. And then the ARM-correction method is conducted on each laboratory-induced ARM to recover a palaeointensity. The recovered palaeointensity is compared with the applied DC field (laboratory field) to estimate if the sample has the ability to recover the applied DC field. If a sample fails the test for a certain ARM, then only palaeointensity values larger than the bias field imparting the ARM are considered valid. The revised criteria were suggested by ref. [56]:

$$E = \frac{W}{L} \quad (1)$$

$$D' = \frac{I - L}{L} \quad (2)$$

where E is the ratio of the 95% confidence interval (W) to the laboratory field (L); D' is the relative error between the retrieved palaeointensity (I) and the laboratory field (L). The test is recognized to

'Pass' if D' lies within the interval of (-0.5 and 1) and if E is <0.5 when D' is negative and <1 when D' is positive. Otherwise, the test will be considered to be 'Fail'.

Following these procedures, we conducted the fidelity test after the NRM AF demagnetization step. Samples were imparted ARMs with varying DC bias fields ranging from 2–50 μT, which are equivalent to TRM fields of ~1.5–37.3 μT if employing a calibration factor of 1.34. They were then stepwise AF demagnetized with intervals of 1–10 mT up to 150 mT. After that, an ARM with a 50 μT DC field was applied to each sample, and they were AF demagnetized with the same interval during each test.

Palaeointensities were calculated with AF demagnetization spectra of the ARMs imparted with various DC fields of 2–50 μT and that of the 50-μT ARM. The corresponding D' and E values were also calculated (Supplementary Table 1). According to the aforementioned criteria, three samples showed good behaviour and are able to record an equivalent TRM field of <3.7 μT (211 and 038) or <7.5 μT (392) while sample 344 was less than ideal, nonetheless a palaeointensity between ~7.5–15 μT could still be retrieved.

1.5 Anisotropy of ARM

Remanence anisotropy sometimes plays an important role in palaeointensity estimation and can potentially introduce additional bias to the results. To estimate this effect, ARM anisotropy measurements were performed on all the studied basalt clasts. An ARM was applied with a peak AF field of 150 mT and a DC bias field of 50 μT along the three orthogonal directions of the samples, following the sequence -z, -x, and -y. A three-axis AF demagnetization step with a peak AF field of 150 mT was inserted before each ARM step.

The calculated anisotropy parameters are summarized in Supplementary Table 2. Anisotropy degree (P) of the samples ranges from 1.13–1.34 while the correction factors derived from matrix calculations of the HC components and the laboratory field directions are generally <~10%. Given the nearly symmetric distribution of the factors centred around 1, no corrections were applied to the final palaeointensity results.

2. VRM and IRM test

During the ~3 to 9-day NRM decay experiment, some of the samples appear quite stable. For example, ~5% of the NRM decayed in ~9 days for sample 211 and ~6% decayed in ~3 days for sample 344 (Supplementary Table 3), indicating NRMs of these samples are less affected by a VRM effect. The other samples show a relatively large VRM portion with ~12% decayed in ~4 days for sample 038 and

~12% decayed in ~3 days for sample 392, indicating these samples may contain a certain amount of fine-grained particles around the superparamagnetic (SP) and single-domain (SD) boundary. A representative sample 392 with the largest decay proportion was selected for the VRM decay and acquisition experiment. Both the amount of VRM acquisition and decay are quite limited (~5%), indicating that a VRM is unlikely able to contaminate the HC components of the samples.

In the IRM test, the NRM of each sample was compared with the IRM acquisition curve to test whether the sample was contaminated by a low-field IRM. The NRM of sample 344 is much weaker than the lowest field of 4 mT, demonstrating it is unlikely to be contaminated by an IRM (Extended Data Fig. 7). The NRM of sample 038 is comparable to an IRM imparted by a 4-mT field, but the AF demagnetization result and the palaeointensity calculated with the LC component (Extended Data Fig. 1, Extended Data Table 1) are able to rule out the possibility that this sample is contaminated by an IRM. The NRM of sample 211 is two times lower than the 9-mT IRM, and the palaeointensity (~96 μT) calculated with the LC by the IRM method is relatively high, such that the LC component cannot be excluded from a low-field IRM contamination. But the effect should be limited and cannot exceed 10 mT according to the AF demagnetization result of the sample (Fig. 3, Extended Data Table 1). The NRM of sample 392 is slightly lower than the 9-mT IRM, and the palaeointensities calculated with the LC by both the ARM and IRM methods are quite high (~220 μT and ~364 μT, respectively; Extended Data Table 1). Combining with the soft behaviour below 10 mT during AF demagnetization (Extended Data Fig. 3), it is highly suspected that the LC component of this sample was contaminated by a low-field IRM at some point during the pretreatment or transportation of the sample. But sample 392 records multiple distinct directions from the LC at AF demagnetization steps >11 mT, excluding the possibility that the HC is affected by IRM contamination.

3. Rock magnetic analysis

3.1 Magnetic susceptibility

The χ_{lf} values for samples 038 and 344 are 6.05 × 10^{-7} m^3/kg and 6.23 × 10^{-7} m^3/kg, respectively, while the χ_{hf} values are 6.03 × 10^{-7} m^3/kg and 6.26 × 10^{-7} m^3/kg, respectively. These values are highly consistent within 1σ. For sample 392, the mean value of χ_{hf} (7.26 × 10^{-7} m^3/kg) is higher than that of χ_{lf} (6.78 × 10^{-7} m^3/kg), coupled with a larger standard deviation, which suggests potential noise interference due to its smaller size (~43.7 mg) compared to the other samples. The χ_{hf} (6.88 × 10^{-7} m^3/kg) of sample 211 is lower than its χ_{lf} (7.13 × 10^{-7} m^3/kg) and shows a statistically significant

difference within 2σ, indicating the possible presence of fine particles close to the SP and SD boundary in the sample. The χ_{lf} data are presented in Extended Data Figure 5a, which fall at the lower end of the published Apollo data range, similar to the Chang'e-5 basalt data.

3.2 Hysteresis loop

All the loop data show strong paramagnetic signals, with magnetizations decreasing to ~5% of their original values after paramagnetic correction (Extended Data Fig. 4a, d, g, j). The samples have low coercivities (B_c) of a few mT and moderate remanent coercivities (B_{cr}) of 29–48 mT. The basalt clasts fall within a broad region on a Day plot[45] with sample 038 close to the Chang'e-5 basalt data and sample 344 deviating the most towards the MD region, indicating a diverse magnetic mineral assemblage with various grain sizes (Extended Data Fig. 5b).

3.3 IRM acquisition and back-field demagnetization

All the samples are generally saturated below ~300 mT with some of them increasing slightly until 500 mT (Extended Data Fig. 4b, e, h, k), indicating a large distribution of the coercivity range. The IRM acquisition curves of samples 211, 038, and 344 were then decomposed using the R package of MAX UnMix (ref. [57]). The IRM data of sample 392 was not analysed because it is noisy. Spectrum analysis reveals the presence of a dominant coercivity component near ~100 mT in all the analysed samples while sample 038 also has a lower component of ~30 mT (Supplementary Fig. 3).

3.4 FORC diagram

All the FORC diagrams display a mixture of SP, SD, and MD signatures (Extended Data Fig. 4c, f, i, l), and the signals along the horizontal axis extend up to ~120 mT, especially for samples 211 and 038, indicating the presence of high-coercivity magnetic particles.

3.5 Magnetic properties of the basalt clasts

Rock magnetic results of the samples, including IRM spectrum analysis, FORCs and projections of the hysteresis parameters on the Day plot, indicate a substantial population of finer SD grains exist in the samples (Supplementary Fig. 3, Extended Data Figs. 4, 5). Combined with the low field values that pass the criteria for the fidelity limit tests (Supplementary Table 1), it indicates that the Chang'e-6 samples have finer metal grain sizes than nearly all Apollo mare basalts, making them good magnetic recorders amongst the lunar samples. The cooling rates calculated by the crystal size of ilmenite in the

basalt clasts in this study vary from ~20–100 °C hr^{-1}, higher than that (<3 °C hr^{-1}) of some reported Apollo mare basalts[2,50], indicating the Chang'e-6 basalts probably cooled faster and thus produced finer metal grain sizes.

4. Microscopic analysis

The X-ray computed tomography results of the clasts show that the basalt clasts maintain original mineral crystalline structures with subophitic or porphyritic textures (Fig. 2), implying that these samples have experienced limited impact reformation. This conclusion is further evidenced by the backscattered electron (BSE) images of the samples. We searched through the chip thin section of sample 038 with porphyritic texture, and did not observe iron grains according to the resolution (~1 μm) of the SEM. Nonetheless, this sample has ideal magnetic-recording capability according to the result of AF demagnetization and palaeointensity fidelity limit test (Extended Data Fig. 1, Supplementary Table 1) and falls into a similar region as the Chang'e-5 basalt clasts on a Day plot (Extended Data Fig. 5b). Referring to the results of Chang'e-5 basalt clasts, it is inferred that sample 038 contains fine iron particles on the hundred-nm scale. Micrometer iron grains were observed in the subophitic clasts (samples 211, 344, and 392), which are much larger than those in sample 038, implying the latter may crystalize faster than the former after eruption.

Raman spectra characteristics of the minerals in the samples can be used for detecting the effect of high-temperature and/or high-pressure metamorphism in some cases. For example, the Raman spectrum from shocked altered plagioclase usually show two broad bands at 480–580 and 900–1,050 cm^{-1} (refs. [58-60]). High-temperature and/or high-pressure shock may cause transition of pyroxene to pyroxene glass which may cause broadening of the Raman bands or generation of high-pressure polymorphs such as majorite or akimotoite[61]. The spectrum of plagioclase in the studied basalt clasts is characterized by peaks at 180, 280, 506, and 990 cm^{-1} (Supplementary Fig. 1), which are recognized as the fundamental Raman vibrations of well-crystallized plagioclase. The well-resolved bands at 314–399, 658–666, and 993–1,008 cm^{-1} for pyroxene also indicate that the mineral structure is unaltered by shock-induced metamorphism[59,62], which is further supported by the lack of high-pressure polymorphs such as majorite or akimotoite in the samples. Therefore, micro-Raman spectra of the main minerals of plagioclase and pyroxene in the Chang'e-6 basalt clasts indicate the characteristic Raman peaks of the minerals are evident and do not show any obviously disturbed crystallinity such as broadening or shifting, further indicating that the studied clasts did not suffer from obvious impact metamorphism.

The plane-polarized and cross-polarized photomicrographs of sample 211 show that the optical extinction of the minerals is sharp (Extended Data Fig. 8). No obvious planar fractures or planar deformation features were observed for plagioclase and pyroxene. Rotating the thin section to different angles does not yield an obvious undulatory extinction among the minerals. These results imply this sample can be categorized as shock level S1 following the classification of refs.[31,63], which indicates the sample has experienced a peak shock pressure of <5 GPa.

5. Possible power source of the lunar dynamo

Core crystallization is currently the most commonly proposed explanation for a long-lived lunar dynamo. However, most models predict a low surface field of ~1–3 μT (refs. [3,10,64]) after ca. 3.5 Ga on the Moon. There is only one model that predicts a maximum surface field of ~4.4 μT by assuming an exclusively dipolar field, which is likely an overestimation[37]. The precession dynamo is also a promising candidate power source, driven by continuous mechanical stirring caused by differential motion between the Moon's core and mantle[36]. But the evolution of the Moon's orbit and the geometry and vigor of the flows in the precession dynamo model are poorly constrained, and the scaling relationship that relates the surface field strength to the input power is largely unknown[10], leaving the generated field strength from this dynamo highly variable as demonstrated by the wide field strength ranges between the different precession models in Supplementary Figure 4. The basal magma ocean (BMO) dynamo model involves the emplacement of a radioactive heat-producing and metalliferous layer at the core-mantle boundary due to the lunar overturn process[34,35] and could significantly expand the dynamo-generating region. Although the electrical conductivity of molten silicate magmas is uncertain by orders of magnitude[34], by assuming high electrical conductivity in the BMO which is considered to be feasible during the mid-early stage of the BMO according to mineralogical experiments[65,66], there is still a possibility that the BMO model achieves a relatively strong surface field of >10 μT (Supplementary Fig. 4).

The moderate high palaeointensities of Chang'e-6 farside basalts at ca. 2.8 Ga stand out among all estimations after ca. 3.0 Ga in the modern lunar dataset and provide a unique constraint on the lunar dynamo operating mechanism. To assess the possible power source generating the Chang'e-6 palaeointensities, we conducted a resampling process based on the Student's *t*-distribution (Methods) on three (211, 038, and 344) of the four samples since the HC component of sample 392 is not origin-trending. The 95% confidence interval of the Chang'e-6 palaeointensity align best with the strong surface magnetic field being produced by a BMO, and the precession dynamo may also serve as a

candidate considering the large uncertainty in this model (Supplementary Fig. 4). However, uncertainties such as potential high fields generated by turbulence effects and possibilities of multiple power sources cannot be ruled out[10]. Our results suggest that the lunar dynamo at mid-early stage was likely powered by a BMO and/or precession dynamo, possibly supplemented by energy from other sources such as core crystallization.

6. Kolmogorov-Smirnov test between the Chang'e-6 palaeointensities and data from 2–1 Ga

The Chang'e-6 basalt clasts yield palaeointensities varying from ~5–21 μT with a median value of ~13 μT at ca. 2.8 Ga while those published data between 2 and 1 Ga vary from ~0.3–5 μT if only considering the reported palaeointensity mean values, indicating there is a high possibility that the former recorded stronger palaeointensities than the latter. However, the possibility that these two datasets overlap with each other to some extent still exists if considering the standard error or calibration constant uncertainty of the data. The variation trend of the lunar magnetic field after ca. 3 Ga thus requires to be further assessed statistically. The 95% confidence interval of the mean palaeointensities (~7–40 μT with a median value of ~15 μT) including both the linear regression error and calibration constant uncertainty for the Chang'e-6 basalt clasts were calculated through a resampling process as described in the Methods section. A similar resampling process was conducted on the Chang'e-5 and Apollo data between 2 and 1 Ga (Fig. 4). The data of the Apollo sample (60255) dated at ca. 1.7 Ga was not included because an upper limit was used in the original work[16]. In generating each Student's *t*-distribution for the Chang'e-5 data, the ARM and IRM palaeointensities of sample 129 were analyzed considering the linear regression error and a 2 standard deviation factor of ~5 for the calibration constants. For the non-heating results of sample 118, only the linear regression error was included because its calibration factors were directly derived from the AI-Shaw method[17]. For the heating-method result of sample 118 as well as the Apollo results (10018 and 15498) derived from the Thellier-Thellier double-heating technique[13,15], only the reported standard error of the palaeointensities was considered.

The resampling datasets for the data between 2 and 1 Ga yielded a 95% confidence interval of ~3–6 μT with a median value of ~4 μT (Supplementary Fig. 4), which differs significantly from those obtained from the Chang'e-6 data (~7–40 μT with a median value of ~15 μT). A Kolmogorov-Smirnov statistical test was then performed on the two resampled datasets to further investigate whether they share the same distribution. The calculated significance value (p) of 0.0 allows us to reject the null hypothesis that these two datasets originate from the same distribution at a confidence level >99%.

Therefore, the statistical analysis indicates that the Chang'e-6 palaeointensities are highly likely to be stronger than those between 2 and 1 Ga.

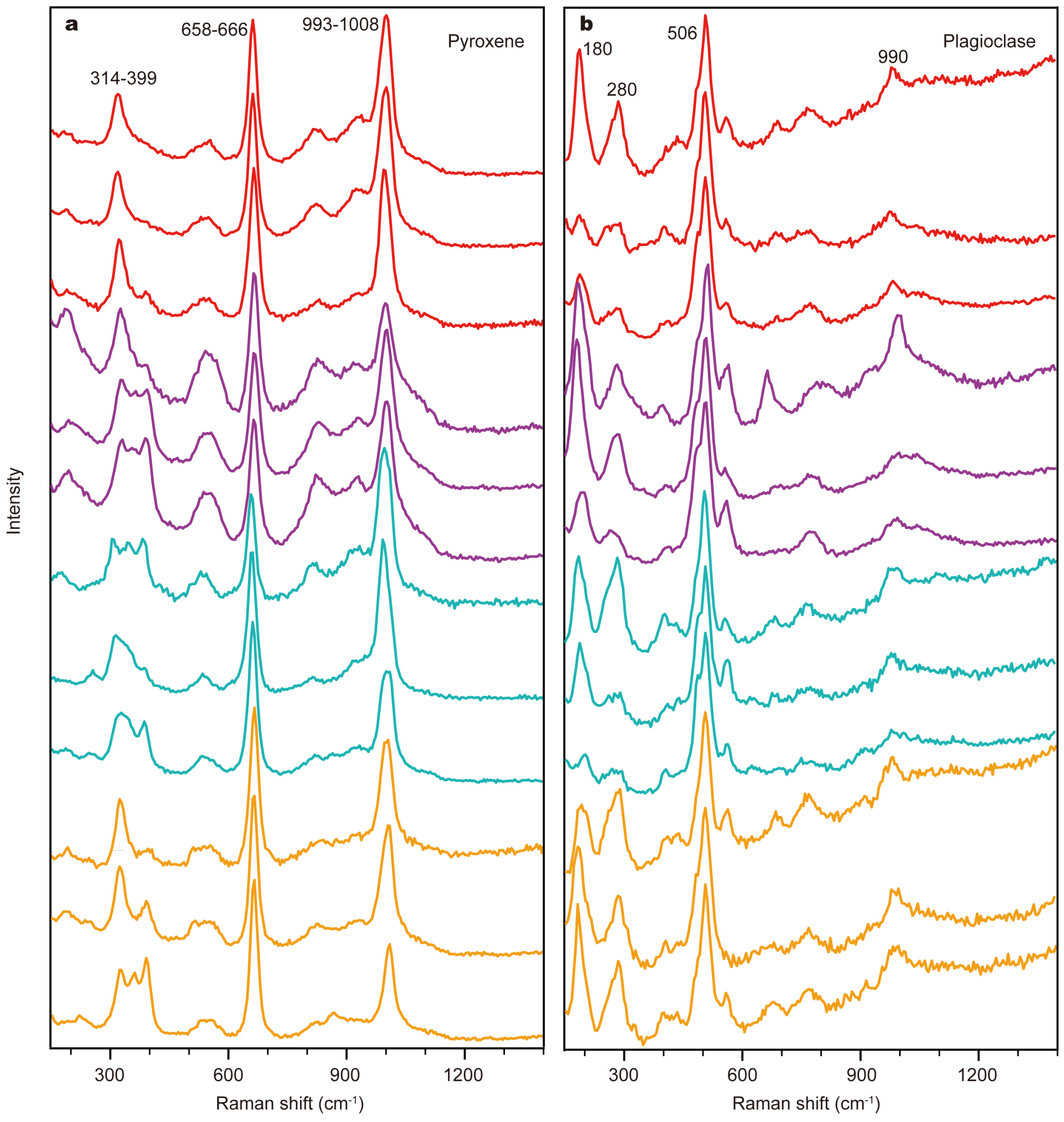

Supplementary Fig. 1 | Raman spectra of pyroxene and plagioclase of the basalt clasts. The spectra of different samples are distinguished by different colours: sample 211 in red, 038 in purple, 344 in cyan, and 392 in yellow.

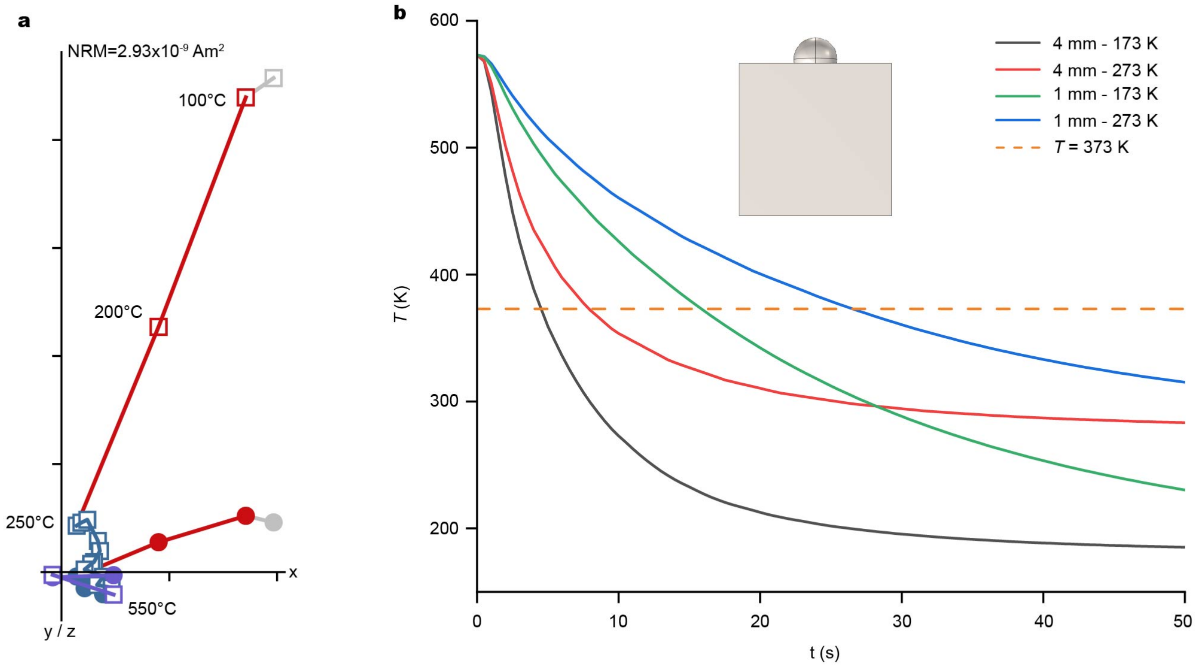

Supplementary Fig. 2 | The thermal demagnetization result of specimen 211b and the correlated cooling time calculation. a, The orthogonal projection plot of thermal demagnetization of specimen 211b. Grey, red, and purple points represent the LT, MT, HT components, respectively. **b,** Cooling time calculations conducted by the COMSOL software. Solid lines with different colours represent models with various ambient temperatures and buried depths. The orange dashed line is the cut-off temperature of 100°C, defined according to the start temperature of the MT component. The insert represents a sketch map of the models.

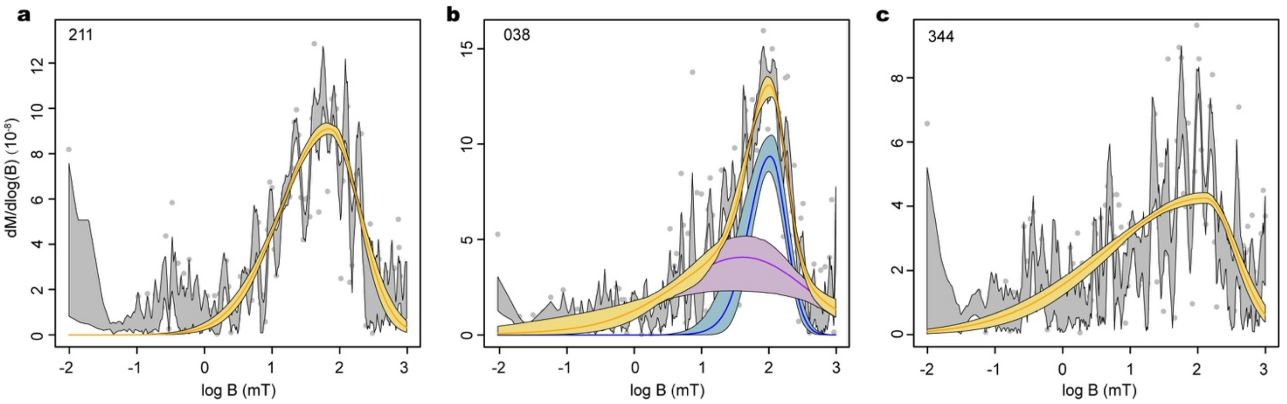

Supplementary Fig. 3 | Coercivity spectrum analysis of the IRM acquisition curves of the Chang'e-6 basalt clasts. Orange line represents the sum of all components. Blue and purple lines represent the high- and low-coercivity component, respectively. Shaded bands represent the 95% confidence intervals of related data. Data were analysed with the web application of MAX UnMix (ref. [57]).

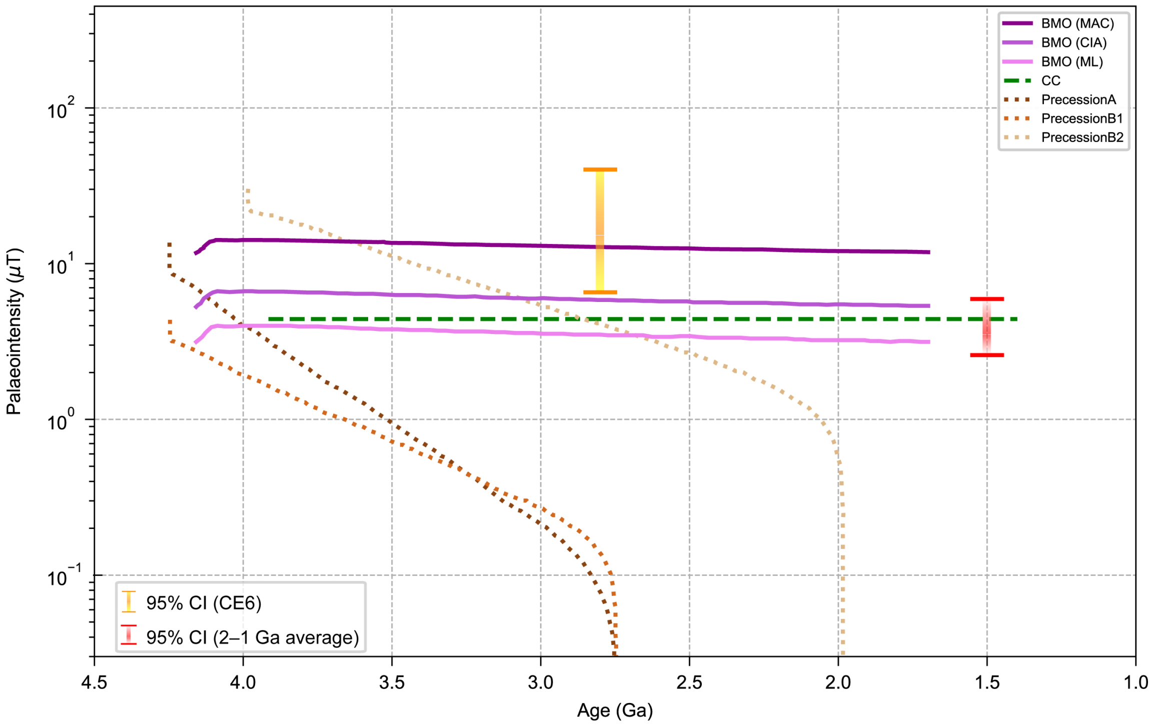

Supplementary Fig. 4 | Predicted strength of the lunar magnetic field from various dynamo models. The basal magma ocean (BMO) models with three different scaling laws used to predict the magnetic field strength are shown: the mixing-length (ML) theory, the balance between Coriolis, inertial, and gravitational (Archimedean) forces (CIA), and the balance between Lorentz, gravitational, and Coriolis forces (magneto-Archimedes-Coriolis, MAC; ref. [34]). Also shown are the precession dynamo models calculated by ref. [36]. Models B1 and B2 here are the nominal model in their Figure S3c and model 4 in their Figure S3b, respectively. The core crystallization (CC) dynamo is from the maximum surface value proposed by ref. [37]. The orange and red error bars represent the 95% confidence interval of the resampled palaeointensities of Chang'e-6 (CE6) and published data between 2–1 Ga, respectively.

Supplementary Table 1 | Statistical parameters of the palaeointensity fidelity limit test of the basalt clasts

Sample	*L* (μT)	*I* (μT)	SE (μT)	*W* (μT)	*E* (%)	*D′* (%)	Result	Equivalent TRM field (μT)
211	2	0.88	0.25	1.02	50.88	-56.21	Fail	1.49
211	5	4.12	0.23	0.93	18.60	-17.60	Pass	3.73
211	10	9.74	0.25	1.01	10.14	-2.55	Pass	7.46
038	2	1.72	0.13	0.52	26.20	-13.82	Pass	1.49
038	5	4.37	0.13	0.52	10.40	-12.56	Pass	3.73
038	10	9.76	0.13	0.54	5.42	-2.37	Pass	7.46
344	5	11.84	3.76	15.35	307.07	136.86	Fail	3.73
344	10	34.20	7.32	29.91	299.05	241.96	Fail	7.46
344	20	29.33	4.00	16.35	81.76	46.67	Pass	14.93
344	50	70.47	7.40	30.24	60.48	40.93	Pass	37.31
392	2	3.78	0.97	3.95	197.26	88.86	Fail	1.49
392	5	1.06	0.46	1.87	37.45	-78.89	Fail	3.73
392	10	8.93	0.61	2.50	24.96	-10.69	Pass	7.46
392	20	16.79	1.20	4.90	24.50	-16.03	Pass	14.93

L, laboratory applied DC field; *I*, retrieved palaeointensity; SE, standard error; *W*, 95% confidence interval of *I*; *E*, the ratio of *W* to *L*; *D′*, the relative error between *I* and *L*.

Supplementary Table 2 | ARM anisotropy parameters of the basalt clasts

Sample	τ_1 (max)	V_1	τ_2 (int)	V_2	τ_3 (min)	V_3	P	T	f_{corr}
211	0.36	(13.21, -3.60)	0.33	(267.90, -76.69)	0.31	(103.98, -12.84)	1.16	0.05	0.90
038	0.36	(307.60, 63.56)	0.33	(139.31, 26.00)	0.32	(227.08, -4.66)	1.13	-0.31	0.98
344	0.36	(258.72, 13.44)	0.33	(110.92, 74.26)	0.31	(170.63, -8.12)	1.16	-0.42	1.04
392	0.37	(80.69, -34.99)	0.35	(60.91, 53.41)	0.28	(163.91, 9.52)	1.34	0.57	1.10

τ_i and V_i, eigenvalues and the corresponding eigenvectors of the anisotropy tensor; P, anisotropy degree; T, anisotropy shape factor with the range of -1–0 for prolate and 0–1 for oblate; f_{corr}, anisotropy correction factor calculated for the HC component.

Supplementary Table 3 | NRM and VRM decay information of the basalt clasts

Sample	NRM_0 (Am^2)	NRM decay amount (Am^2)	NRM after decay (Am^2)	NRM decay rate (Am^2/log(s))	NRM decay (%)	NRM decay time (day)
211	6.06E-09	3.01E-10	6.32E-09	5.13E-11	4.97	8.8
038	4.58E-10	5.41E-11	5.02E-10	9.74E-12	11.81	4.2
344	3.15E-10	1.90E-11	3.22E-10	3.50E-12	6.03	3.2
392	5.85E-10	7.12E-11	5.80E-10	1.31E-11	12.17	3.1
**Sample**	**VRM acquire (Am^2)**	**VRM acquire rate (Am^2/log(s))**	**estimated VRM return to Earth (Am^2)**	**time exposure to Earth's field**	**VRM acquire (%)**	**VRM acquire time (day)**
392	2.77E-11	4.81E-12	3.23E-11	2 months	4.78	6.7
**Sample**	**VRM_0 (Am^2)**	**VRM decay (Am^2)**	**VRM after decay (Am^2)**	**VRM decay rate (Am^2/log(s))**	**VRM decay (%)**	**VRM decay time (day)**
392	5.58E-10	3.03E-11	5.52E-10	4.81E-12	5.42	2.5

Supplementary Table 4 | Angular difference between the MC and HC direction of the basalt clasts

Sample	MC direction	HC direction	Angular difference (°)
211	[82.6°, -42.1°]	[96.4°, -26.4°]	19.35
038	[57.4°, 46.1°]	[50.8°, -59.3°]	105.54
344	[169.1°, 40.4°]	[179.8°, 38.7°]	8.42
392	[172.8°, 47.7°]	[314.6°, -56.9°]	155.29

Supplementary Table 5 | Compiled lunar palaeointensities from Apollo returned samples

Sample	Age (Ga)	B_{anc} (μT)	σ_B_{anc} (μT)	Method	References	Note
10020	3.72 ± 0.04	66	± 37 (reported)	IRM	Ref. [2]	
10017	≤3.56	67	± 15 (reported)	ARM	Ref. [12]	IRM used
		71	± 21 (reported)	IRM		
10049	3.336 ± 0.008	65	± 14 (reported)	ARM	Ref. [12]	IRM used
		77	± 18 (reported)	IRM		
10018	1.542 ± 0.019	1.24	± 0.2 (reported)	double heating	Ref. [15]	
12002	3.26 ± 0.06	≈50	-	IRM	Ref. [67]	not used
12017 (basalt)	3.345 ± 0.005	<37	-	fidelity limit	Refs. [33,50,68]	
12017 (glass)	<0.007	<7	-	fidelity limit	Refs. [33,50,68]	not used
12022	3.194 ± 0.0025	<4	-	fidelity limit	Ref. [33]	
12015	3.169 ± 0.004	<4	-	fidelity limit	Ref. [7]	
12009	3.163 ± 0.005	<7	-	fidelity limit	Ref. [7]	
12008	3.065 ± 0.009	<7	-	fidelity limit	Ref. [7]	
15597	3.3 ± 0.2	<7	-	fidelity limit	Ref. [33]	
15016	3.281 ± 0.008	<37	-	fidelity limit	Refs. [33,50,69]	
15556	3.233 ± 0.007	<75	-	fidelity limit	Refs. [33,50,69]	
15498	1.32 ± 0.43<br>2.5-1.0<br>1.47 ± 0.45	5	± 2 (reported)	Thellier-IZZI	Refs. [13,14,70]	
15015	0.91 ± 0.11	<0.08	-	AREMc	Ref. [14]	
15465	0.44 ± 0.01	<0.06	-	AREMc	Ref. [14]	
64455	0.002	11.8-28.4	-	TTRM, Thellier-Coe, IRM	Ref. [19]	not used
60015	3.46 ± 0.05	<5	-	controlled TRM experiment	Refs. [33,71]	not used
61195	3.41 ± 0.43	4.9	± 2.5 (reported)	double heating	Ref. [16]	

60019	3.35 ± 0.43	1.1	± 2.3 (reported)	double heating	Ref. [16]	
60255	1.70 ± 0.43	0.3	± 0.5 (reported)	double heating	Ref. [16]	
76535	4.249 ± 0.012	23	± 12 (reported)	ARM	Refs. [11,72]	IRM used
		40	± 10 (reported)	IRM		
71505	3.7 ± 0.02	95	47.5-190 (set)	IRM	Refs. [14,67,73]	
71567	3.75 ± 0.01	111	55.5-222 (set)	IRM	Refs. [14,67,73]	
70017	3.7 ± 0.1	42	21-84 (set)	IRM	Refs. [67,74]	
75035	3.753 ± 0.009	50.7	± 13.5 (reported)	ARM	Ref. [34]	
75055	3.752 ± 0.009	57	± 17.3 (reported)	ARM	Ref. [34]	
10003	3.91 ± 0.03	14.62	± 9.29 (reported)	ARM	Ref. [75]	not used (fidelity failed)
		54.10	± 4.66 (reported)	IRM		
10044	3.73 ± 0.05	12.24	± 7.13 (reported)	ARM	Ref. [75]	not used (potential shocked)
		9.73	± 18.82 (reported)	IRM		
10069	3.54 ± 0.01	79.91	± 12.69 (reported)	ARM	Ref. [75]	IRM used
		43.00	± 8.68 (reported)	IRM		
10071	3.51 ± 0.06	12.72	± 2.88 (reported)	ARM	Ref. [75]	IRM used
		8.65	± 3.03 (reported)	IRM		

This is an updated version of Table S1 in ref. [17] and detailed descriptions about the data can be found there. Four new published data from ref. [75] were added here.

46. Weiss, B. P., Gattacceca, J., Stanley, S., Rochette, P. & Christensen, U. R. Paleomagnetic records of meteorites and early planetesimal differentiation. *Space Sci. Rev.* **152**, 341–390 (2009).

47. Stephenson, A. Three-axis static alternating field demagnetization of rocks and the identification of natural remanent magnetization, gyroremanent magnetization, and anisotropy. *J. Geophys. Res. Solid Earth* **98**, 373–381 (1993).

48. Acton, G. *et al.* Micromagnetic coercivity distributions and interactions in chondrules with implications for paleointensities of the early solar system. *J. Geophys. Res. Solid Earth* **112**, B03S90 (2007).

49. Marsh, B. D. Crystal size distribution (CSD) in rocks and the kinetics and dynamics of crystallization. *Contrib. Mineral. Petrol.* **99**, 277–291 (1988).

50. Tikoo, S. M. *et al.* Magnetic fidelity of lunar samples and implications for an ancient core dynamo. *Earth Planet. Sci. Lett.* **337**, 93–103 (2012).

51. Chowdhary, S. K., Collinson, D. W., Stephenson, A. & Runcorn, S. K. Further investigations into lunar palaeointensity determinations. *Phys. Earth Planet. Inter.* **49**, 133–141 (1987).

52. Narrett, I. S. *et al.* Lunar crustal magnetization from impact-generated plasma amplification of the lunar dynamo. *55th Lunar and Planetary Science Conference*, **1515** (2024).

53. Hopper, R., Onorato, P. & Uhlmann, D. Thermal histories and crystal distributions in partly devitrified lunar glasses cooled by radiation in *Lunar Science Conference, 5th, Houston, Tex., March 18–22, Proceedings. Volume 3.(A75-39540 19–91) New York, Pergamon Press, Inc.* 2257–2273 (1974).

54. Ivanov, M. A. *et al.* Geologic history of the northern portion of the South Pole-Aitken basin on the Moon. *J. Geophys. Res.: Planets* **123**, 2585–2612 (2018).

55. Yue, Z. *et al.* Geological context of the Chang'e-6 landing area and implications for sample analysis. *Innovation (Camb)* **5**, 100663 (2024).

56. Bryson, J. F. J., Weiss, B. P., Harrison, R. J., Herrero-Albillos, J. & Kronast, F. Paleomagnetic evidence for dynamo activity driven by inward crystallisation of a metallic asteroid. *Earth Planet. Sci. Lett.* **472**, 152–163 (2017).

57. Maxbauer, D. P., Feinberg, J. M. & Fox, D. L. MAX UnMix: A web application for unmixing magnetic coercivity distributions. *Comput. Geosci.* **95**, 140–145 (2016).

58. Fritz, J., Greshake, A. & Stöffler, D. Micro-Raman spectroscopy of plagioclase and maskelynite in Martian meteorites: Evidence of progressive shock metamorphism. *Antarct. Meteorite Res.* **18**, 96–116 (2005).

59. Acosta-Maeda, T. E., Scott, E. R. D., Sharma, S. K. & Misra, A. K. The pressures and temperatures of

meteorite impact: Evidence from micro-Raman mapping of mineral phases in the strongly shocked Taiban ordinary chondrite. *Am. Mineral.* **98**, 859–869 (2013).

60. Chen, M. & El Goresy, A. The nature of maskelynite in shocked meteorites: not diaplectic glass but a glass quenched from shock-induced dense melt at high pressures. *Earth Planet. Sci. Lett.* **179**, 489–502 (2000).

61. Feng, L. *et al.* Shock-induced P-T conditions and formation mechanism of akimotoite-pyroxene glass assemblages in the Grove Mountains (GRV) 052082 (L6) meteorite. *Am. Mineral.* **102**, 1254–1262 (2017).

62. Stähle, V., Altherr, R., Nasdala, L. & Ludwig, T. Ca-rich majorite derived from high-temperature melt and thermally stressed hornblende in shock veins of crustal rocks from the Ries impact crater (Germany). *Contrib. Mineral. Petrol.* **161**, 275–291 (2010).

63. Stöffler, D., Hamann, C. & Metzler, K. Shock metamorphism of planetary silicate rocks and sediments: Proposal for an updated classification system. *Meteorit. Planet. Sci.* **53**, 5–49 (2018).

64. Hamid, S. S., O'Rourke, J. G. & Soderlund, K. M. A long-lived lunar magnetic field powered by convection in the core and a basal magma ocean. *Planet. Sci. J.* **4**, 88 (2023).

65. Yoshino, T., Walter, M. J. & Katsura, T. Connectivity of molten Fe alloy in peridotite based on in situ electrical conductivity measurements: implications for core formation in terrestrial planets. *Earth Planet. Sci. Lett.* **222**, 625–643 (2004).

66. Pommier, A., Walter, M. J., Hao, M., Yang, J. & Hrubiak, R. Acoustic and electrical properties of Fe-Ti oxides with application to the deep lunar mantle. *Earth Planet. Sci. Lett.* **628** (2024).

67. Cournède, C., Gattacceca, J. & Rochette, P. Magnetic study of large Apollo samples: Possible evidence for an ancient centered dipolar field on the Moon. *Earth Planet. Sci. Lett.* **331**, 31–42 (2012).

68. Buz, J. *et al.* Magnetism of a very young lunar glass. *J. Geophys. Res.: Planets* **120**, 1720–1735 (2015).

69. Shuster, D. L. & Cassata, W. S. Paleotemperatures at the lunar surfaces from open system behavior of cosmogenic ^{38}Ar and radiogenic ^{40}Ar. *Geochim. Cosmochim. Acta* **155**, 154–171 (2015).

70. Fagan, A. L., Joy, K. H., Bogard, D. D. & Kring, D. A. Ages of globally distributed lunar paleoregoliths and soils from 3.9 Ga to the present. *Earth Moon Planets* **112**, 59–71 (2014).

71. Lawrence, K., Johnson, C., Tauxe, L. & Gee, J. Lunar paleointensity measurements: Implications for lunar magnetic evolution. *Phys. Earth Planet. Inter.* **168**, 71–87 (2008).

72. Garrick-Bethell, I., Weiss, B. P., Shuster, D. L., Tikoo, S. M. & Tremblay, M. M. Further evidence for early lunar magnetism from troctolite 76535. *J. Geophys. Res.: Planets* **122**, 76–93 (2017).

73. Stöffler, D. *et al.* Cratering history and lunar chronology. *Rev. Mineral. Geochem.* **60**, 519–596 (2006).

74. Schmitt, H. H. *et al.* Revisiting the field geology of Taurus–Littrow. *Icarus* **298**, 2–33 (2017).

75. Jung, J.-I., Tikoo, S. M., Burns, D., Váci, Z. & Krawczynski, M. J. Assessing lunar paleointensity variability during the 3.9 - 3.5 Ga high field epoch. *Earth Planet. Sci. Lett.* 638, 118757 (2024).
